# Therapeutic Metallic Ions in Bone Tissue Engineering: A Systematic Review of The Literature

**DOI:** 10.22037/ijpr.2020.112641.13894

**Published:** 2019

**Authors:** Hannaneh Safiaghdam, Hanieh Nokhbatolfoghahaei, Arash Khojasteh

**Affiliations:** a Student Research Committee, Dental school, Shahid Beheshti university of medical sciences, Tehran, Iran.; b Department of Tissue Engineering and Applied Cell Sciences, School of Advanced Technologies in Medicine, Shahid Beheshti University of Medical Sciences, Tehran, Iran.; c Dental Research Center, Research Institute of Dental Sciences, Shahid Beheshti University of Medical Sciences, Tehran, Iran.

**Keywords:** Bone tissue engineering, Metallic ions, Scaffolds, Therapeutic ions, Dug delivery

## Abstract

An important field of bone tissue engineering (BTE) concerns the design and fabrication of smart scaffolds capable of inducing cellular interactions and differentiation of osteo-progenitor cells. One of these additives that has gained growing attention is metallic ions as therapeutic agents (MITAs). The specific biological advantage that these ions bring to scaffolds as well as other potential mechanical, and antimicrobial enhancements may vary depending on the ion entity, fabrication method, and biomaterials used. Therefore, this article provides an overview on current status of *In-vivo *application of MITAs in BTE and the remaining challenges in the field. Electronic databases, including PubMed, Scopus, Science direct and Cochrane library were searched for studies on MITAs treatments for BTE. We searched for articles in English from January-2000 to October-2019. Abstracts, letters, conference papers and reviews, *In-vitro* studies, studies on alloys and studies investigating effects other than enhancement of new bone formation (NBF) were excluded. A detailed summary of relevant metallic ions with specific scaffold material and design, cell type, animal model and defect type, the implantation period, measured parameters and obtained qualitative and quantitative results is presented. No ideal material or fabrication method suited to deliver MITAs can yet be agreed upon, but an investigation into various systems and their drawbacks or potential advantages can lead the future research. A tendency to enhance NBF with MITAs can be observed in the studies. However, this needs to be validated with further studies comparing various ions with each other in the same animal model using critical-sized defects.

## Introduction

Successful induction of bone tissue regeneration is a complicated process requiring harmonic interplay of cells, cell supporting scaffolds and bioactive materials (1). An important field of bone tissue engineering (BTE) concerns the design and fabrication of smart scaffolds capable of inducing cellular interactions and differentiation of osteo-progenitor cells. This can be achieved by loading the engineered scaffold with various therapeutic agents that give the scaffold a dual function: as a bed for new tissue growth and as a carrier for controlled in-situ drug delivery (2). One of these additives that has gained growing attention is metallic ions as therapeutic agents (MITAs). As it has been shown in the recent literature that some ions are able to guide the differentiation of stem cells into a desired path, there is great hope in employing them in regenerative medicine (3-5). MITAs have essential roles in body as cofactors of various enzymes, in cellular metabolism, signaling pathways, ionic channels and other biologic procedures (6). Metallic ions enhance osteogenic differentiation of mesenchymal stem cells & regulate osteoclast-mediated bone resorption. Pathways known to be involved in osteogenic differentiation such as Wnt signaling has been reported to be influenced by stimulation with trace ions like lithium, magnesium, strontium, or zinc (7). Other related osteogenic markers such as Runt-related transcription factor 2 (Runx2), osteonectin, osteopontin, and collagen type one are also enhanced with addition of metallic ions (8-12). MITAs also have a role in promoting differentiation, migration and capillary formation of endothelial cells as well as inducing secretion of pro-angiogenic factors such as vascular endothelial growth factor (VEGF) (13-15). Inorganic ions are also bacteriostatic which give the scaffold impunity against bacterial adhesion & infection which disturb tissue integration (16, 17). Moreover, MITAs have relatively lower risk of cancer compared with recombinant proteins or genetic modifications (18). The bioactivity of a bone scaffold depends on the interaction of its constituent molecules with stem cells and pre-osteoblasts at the interface (19). Along with the growth factor proteins such as bone morphogenetic protein (BMP) family or other peptides and small molecules, research has been focused on metallic ions (20-22). Their prominent advantages against growth factors and other organic drugs are the lower expense, relative stability during fabrication procedure and higher function in lower concentrations (21, 23). Local delivery of these metal ions compared to taking them via oral routes has the advantage of better control over dose and distribution of the drug (24). Moreover, the ionic state of a few metallic ions is unstable and may cause toxic effects in case of direct ingestion. In case of systemic distribution, non-specific adverse effects in neurologic, cardiologic, hematologic or endocrine systems may be observed (25, 26). It should be noted that designing a scaffold that regulates the specific amount of ion released in a particular period is necessary to prevent local toxic effects and ion’s side effect on the metabolism of adjacent cells (27). Modulating release kinetics of ions from scaffold in a controlled manner, reduces the accumulation of ion and dose-dependent toxicity and results in induction of favorable cell behavior (3). Different methods such as Ion exchange, solvent casting, salt leaching, electrospinning, three dimensional (3D) printing, freeze-drying, and laser sintering have been applied in fabrication of bone scaffolds incorporating MITAs (28-33). These inorganic ions can be incorporated into various materials such as bioactive glasses, glass ceramics, calcium phosphates, hydroxyapatite (HA), alpha and beta-tricalcium phosphates, biodegradable polymers and composite scaffolds. This addition alters degradation behavior, mechanical characteristics and biological function of scaffolds (34). In this systematic review, we aimed to analyze *In-vivo *studies on MITAs less commonly applied in BTE and present an overview upon their efficacy in enhancing bone regeneration. 

## Experimental

**Figure 1:**
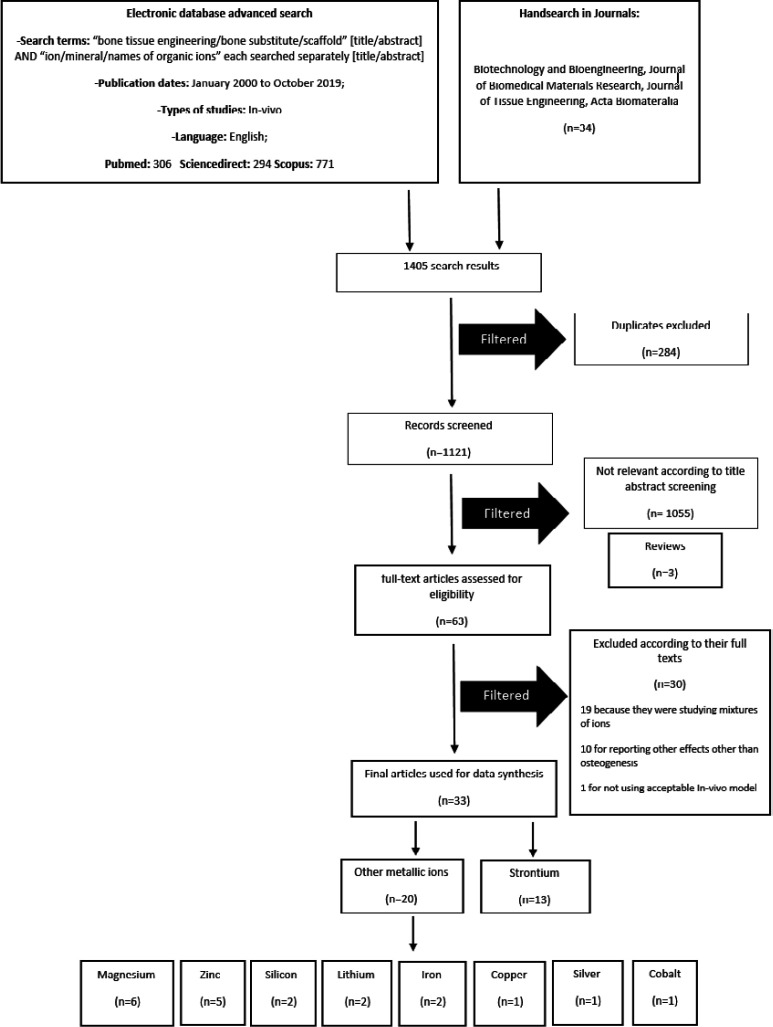
PRISMA Flowchart

This study has been designed and conducted according to the preferred reporting items for systematic reviews and meta-analyses (PRISMA) guidelines (35). Electronic databases, including PubMed, Scopus, Science direct and Cochrane library were searched for studies on MITAs treatments for BTE. The following keywords were used: bone tissue engineering/bone substitute/scaffold [title/abstract] AND ion/mineral/names of organic ions each searched separately [title/abstract]. We searched for articles in English from January 2000 to October 2019 and checked the reference list of related reviews and the following journals for additional relevant studies: (1) Biotechnology and Bioengineering; (2) Journal of Biomedical Materials Research; (3) Journal of Tissue Engineering; (4) and Acta Biomateralia. A total of 1405 articles were collected. Eligibility checking and data extraction were performed independently by two reviewers. Any disagreements were resolved by discussion. Inclusion and exclusion criteria of the study were applied through the initial screening of titles and abstracts. Total of 284 duplicate results were excluded. Abstracts, letters, conference papers, and reviews were excluded (n=3). Abstracts and titles were screened and 1055 articles were excluded as they were *In-vitro*. Full-texts for the remainder were obtained (n = 63). Studies on alloys & mixtures of ions (n=19) were excluded because the osteoinductive activity could not be completely attributed to the one specific component. Also studies investigating ion effects other than enhancement of new bone formation (NBF) (n = 10) were excluded. One study was excluded because of the unacceptable *In-vivo* model. Figure 1 shows a diagram of study selection process.

The included studies were screened for the scaffold and fabrication method, cell type used, the animal model and size & location of the defect, the implantation period, study groups, measured parameters and obtained qualitative and quantitative results. A meta-analysis could not be conducted due to the heterogeneity of the data.

## Results

A total of 33 articles met our inclusion and exclusion criteria and were reviewed. The data is summarized in Table 1. Studies were classified according to the metallic ion added to the scaffolds. Strontium was the most studied ion, with 13 *In-vivo* studies (12, 36-47), followed by magnesium with 6 (9, 30, 48-51) and zinc with 5 articles (22, 52-55). Two articles were found on silicon (10, 56), lithium (8, 57) and iron (58, 59) each, and copper (60), silver (61), and cobalt (62, each had only one studies. 

These metallic ions were incorporated into various scaffolds: A total of 11 articles used polymers such as poly-caprolactone (PCL) (n = 5) (10, 37, 38, 48, 51), poly lactic-co-glycolic acid (PLGA) (n=2) (9, 30), Poly-L-lactic acid (PLLA) (n = 2) (39, 61), chitosan (60), and combination of collagen and alginate (62). Among the 13 studies which used ceramics, six used hydroxyapatite (HA) (8, 46, 49, 53, 56, 58), four used calcium polyphosphates (CPP) (43-45, 57), three used tricalcium phosphate (TCP) (22, 54, 55). One study used magnesium phosphate 2D nano-sheets (50). Another ten studies evaluated the efficacy of ions in composite scaffolds. HA was used in combination with collagen in 3 studies (47, 52, 53), with poly(γ-benzyl-l-glutamate) (PBLG) in 2 studies (40, 63), with PCL in 2 other studies (36, 61), and with PLLA in one study (41). Collagen polymer was combined with amorphous calcium phosphate porous microspheres in a study on strontium ion (52).

Only six studies implanted the designed scaffolds with stem cells to enhance bone formation. These included rabbit adipose derived stem cells (ADSCs) (40), rabbit bone marrow derived stem cells (BMSCs) (45), mice ADSCs (42), human telomerase immortalized BMSCs (37), hypoxia preconditioned BMSCs (8), and finally MC3T3e1 (mouse pre-osteoblast) cell lines (10). 

Most frequently used animal model was rat (n=17) followed by rabbit (n=12). Two studies used mice while one study reported beagle dog (30) and another reported goat (64) as its animal model. 

The most commonly used defect model was a critical sized calvarial defect. Two studies investigated NBF in 8 mm defects (9, 61) while seven used 5 mm defects (12, 36, 39, 46, 56, 60, 62). A 15 mm defect was used in a goat model calvarium (49), as well as a 10 mm rabbit calvarial defect (43) and a 2mm mouse cranial defect (38). One infected cranial defect was used to evaluate the antimicrobial effect of silver along with osteoinductivity (61). Eleven femoral (22, 40-42, 44, 51-53, 55, 58, 59), five tibial (8, 47, 50, 57, 65) and one radial defects (45) were also studied in varying dimensions considered to be critical. Subcutaneous implantation was applied in 3 studies (10, 37, 48) and one study reported a split mouth socket preservation in a dog model (30).

Average implantation period was 11.27 weeks which varied between one study evaluating NBF after 2 weeks (50) and another long-term study waiting for a maximum of 60 weeks (55). However, most of the studies (n=11) had a 12-week implantation period, followed by 10 studies having an 8-week period. 

Different parameters including X-ray radiography or computed tomography (CT) measured bone mineral density, micro-CT derived new bone volume, mechanical testings, Dual x-ray absorptiometry, histomorphomteric analysis with Haemotoxylin and Eosin (H&E) and Goldner-Masson’s trichrome staining, quantitative real time polymerase chain reaction (qRT-PCR) measurement of different RNAs and western blot of the resultant proteins were evaluated. Other stainings such as von kossa for calcium detection, toluidine blue for cell nuclei, acid fuschin, fast green, sirius red for collagen, tartrate-resistant acid phosphatase for osteoclast and giemsa for osteoblast as well as chloroacetate esterase for neutrophils and human vimentin antibody staining for endothelium was conducted in some studies. Immunohistochemistry analysis helped in identification of various osteogenic markers such as osteonectin, osteopontin, collagen type I, β-catenin as well as angiogenic factors such as vascular endothelial growth factors (VEGF), basic fibroblast growth factor (BFGF). 

**Figure 2:**
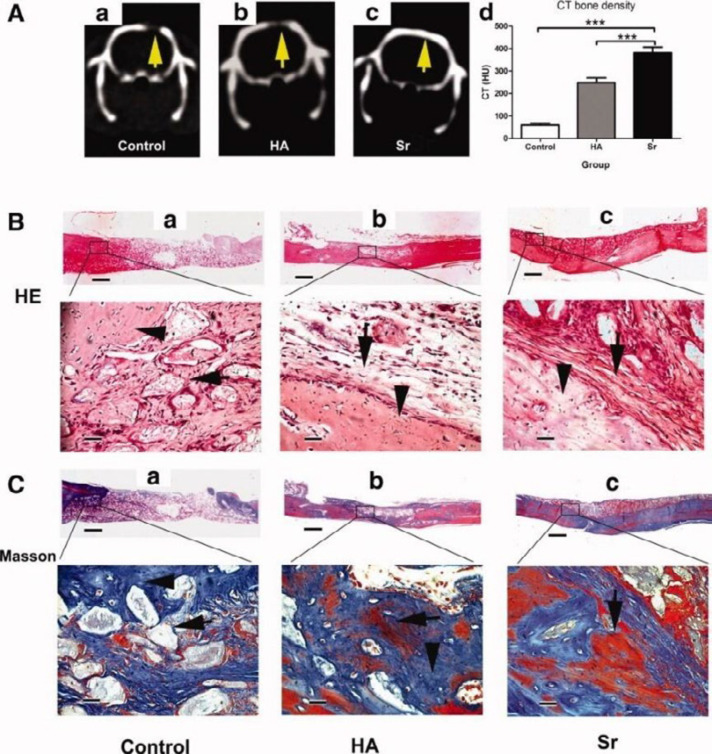
CT scanning and histological analysis of bone formation at 3 months after the transplantation. (A): Representative radiographic analysis of bone formation in the control (a), hydroxyapatite (HA) (b), and strontium (Sr) groups (c). (B): Representative histological analysis (H&E staining) of bone formation in the control (a), HA (b), and Sr group (c). (C): Representative histological analysis (Masson staining) of bone formation in the control (a), HA (b), and Sr group (c). Scale bar = 800 μm (B, C, low magnification); = 75 μm (B, C, high magnification). ***, p < .001. Abbreviations: CT, computed tomography; HA, hydroxyapatite; HU, Hounsfield unit; Sr, strontium (46). - The images are provided with permission from Stem Cells Publications, John Wiley & Sons Publication group (license number: 4755280758795)

**Table 1:** Metallic ions other than strontium used in bone tissue engineering by order of frequency and published year

Author (year)	Ion	scaffold	Ion addition method	Cell type	Animal model	Defect size & location	Implantation period	Groups		Parameters	Observed effect	Ref
Wei (2018)	Ag	PLLA + nano silver + polydopamine + PEG	W1/O/W2 double emulsion method	-	SD rat	Infected 8mm critical-sized calvarial defect	8 W	A. PEG10% +polydopamine + PLLA B.PEG% + polydopamine + PLLA + Ag C. Empty		H&E *In-vitro* incubation of granulation tissues to observe the bacterial growth	The Ag group showed much reduced infection in comparison to other groups NBF was higher in Ag	(61)
Perez (2015)	Co	Collagen + BMP2 + alginate + Co	Crosslinking	-	male SD rat	5 mm critical sized calvarial defects	6 W	A. no Co/BMP2, B. Co only C. BMP2 only D. Co/BMP2 both		µ-CT H&E	Most calcified tissue type in Co with BMP2 Bone volume: Co only (11.30 ± 2.00 %) < bare (14.51 ± 2.45 %) < BMP2 only (29.94 ± 2.53 %) < Co with BMP2 (35.10 ± 2.81 %)	(62)
D’Mello (2014)	Cu	Cu + chitosan	Freeze-drying	-	Male Fisher 344 rat	5 mm critical sized calvarial defect	4 W	A. empty B. chitosan C. Chitosan + Cu		µ-CT H&E	NBF increased with copper BV/TV: copper-chitosan= 11x empty defects and 2x chitosan scaffolds	(60)
Russo (2017)	Fe	HA + Magnetite (90-10%)	Homogeneous mixed slurry	-	Male rabbit	6x8 mm Critical size femoral defect	4,12 W	A. HA B. HA + Magnetite		Toluidine Blue Acid Fuchsin Fast Green TRAPP	NBF% 4^th^ week: A: 35 ± 4 B: 19 ± 4 NBF 12^th^ week: A: 29 ± 6 B: 15± 5	(58)
De Santis 2015)	Fe	PCL + HA + Fe (80-20%)	Mixing and using ultrasonic bath	-	Male rabbits (Oryctolagus cuniculus)	6x8 mm Critical size femoral defect	4W	A.PCL B. PCL/FeHA		Toluidine Blue Acid Fuchsin Fast Green	A similar to B	(59)
Ma (2018)	Li	CPP + Li	chemical precipitation	-	adult male Japanese white rabbits	Bilateral 3x5 mm tibial defect	4, 8 W	A. 0.1%LiCPP B. 1.0%LiCPP C. 2.0%LiCPP D. 3.0%LiCPP E.CPP		H&E	NBF: C>B>E	(57)
Li (2018)	Li	HA + Li	chemical precipitation	BMSCs	male Japanese white rabbits	5 mm diameter tibial defect	6, 12 W	A. 1.5% Li- HA + hypoxic BMSCs B. 1.5%Li- HA + normal BMSCs C. 1.5%Li-HA D. HA		µ-CT H&E MT IHC qRT-PCR Western blot	BV/TV%: A>B>C>D NBF: A>B>C>D	(8)
Yuan (2018)	Mg	PLGA + MgO + MgCO3	W1/O/W2 double emulsion method	-	female SD rat	8 mm critical size calvarial defect	4,8,16 W	A. PLGA B. PLGA + MgO + MgCO3 in 1:1 C. Empty		µ-CT H&E MT IHC	BV/TV B: 32.9 ± 5.6 % A: 8.1 ± 2.5 % BMD B: 325.7 ± 20.2 A: 124 ± 35.8 Higher cell infiltration and NBF & Higher OCN and OPN In B	(9)
Suryavanshi (2017)	Mg	PCL + MgO 10%	precipitation-calcination method	-	SD rat	subcutaneous implantation	2, 4, 8 W		A. PCL B.PCL + Mgo	H&E	No NBF Proved biocompatibility	(48)
Deng (2017)	Mg	HA + Mg + rhBMP2	chemical precipitation	-	Goat	Bi-parietal 15 mm calvarial defect	4,8,12 W	A. HA + Mg + rhBMP-2 B. HA + Mg C. HA		H&E MT CT qRT-PCR	Gray value & VEGF and Col1 & Mean osteogenic area: A>B>C	(49)
Laurenti (2016)	Mg	2D Magnesium Phosphate Nanosheets	percipitation method	-	Rat	Tibia defect	3 days 1,2 W	A. Nano MgP B. empty		µ-CT H&E qRT-PCR	NBF: MgP > empty Defect fully filled at 14days Runx2 and Col1 expressed more at day3 in MgP Upregulation of osteoclasts	(50)
Brown (2015)	Mg	PLGA + Mg	Solvent casting, salt leaching	-	Beagle dog	Split mouth socket preservation	8,16 W	A. PLGA + 10 mg Mg B. Empty		µ-CT MT Von kossa staining Chloroacetate esterase staining	Defect height 8^th^ week A:1.9 B:2.2mm Defect height 16^th^ week A:0.9 B:1.6 BV/TV 8^th^ week: A:29% B:21% BV/TV 16^th^ week: A:35% B:31% No sign of chronic inflammation	(30)
Wong (2014)	Mg	PCL + Mg	Salt leaching	-	Female SD rat	Bilateral 2x6 mm femoral defect	12 W	A. PCL B. Silane coupled Mg-PCL		Giemsa Staining	NBF & osteoblast B>A	(51)
Wang (2018)	Si	PCL + nano Si (0%, 1%, 5%, and 10% w/w )	Mixing and using ltrasonic bath	MC3T3 e1 cells	female & male SD rats	Subcutaneous implantation	1,4 W	A. 1.0% nano-Si PCL B. 10% nano-Si PCL C. PCL		H&E MT IHC	Higher calcification & Higher OCN In B	(10)
Cui (2016)	Si	Si-HA + BMP-2-related peptide (P28)	Freeze-drying	-	male SD rat	5 mm critical size calvarial defect	6, 12 W	A. empty B. Si/HA C. Si/HA + P28 D.Si/HA + rhBMP2		µ-CT H&E	NBF 6^th^ week A: 9.44 ± 2.20% B: 16.22 ± 2.31% C: 49.99 ± 5.51% D: 51.15 ± 3.46% NBF 12^th^ week: A: 21.94 ± 1.86% B: 36.39 ± 3.57% C: 84.18 ± 6.03% D: 86.63 ± 3.82% BV/TV: C=D>B>A	(56)
Samanta (2019)	Zn	βTCP +5% Zn or Mg or Ti	aqueous solution combustion technique	-	rabbits	5 ×2.5 ×3 mm Femoral defect	4,8 W	A. Empty B. βTCP C. βTCP + Zn D. βTCP + Mg E. βTCP + Ti		µ-CT X-ray radiography H&E SEM IHC	NBF 1^st^ month: A: 16.39 ± 1.9 B: 27.48 ± 1.9 C: 35.64 ± 2.0 D: 41.35 ± 2.0 E: 50.31 ± 1.9 NBF 2^nd^ month: A: 30.53 ± 2.0 B: 34.24 ± 2.0 C: 48.40 ± 2.0 D: 50.55 ± 2.0 E: 65.06 ± 3.0	(22)
Yu (2017)	Zn	HA/COL + Zn	lyophilization fabrication	-	male SD rat	3.5x4 mm critical size femoral condyle defect	8 W	A. HA/Col B. HA/COL + Zn Zn/(Zn + Ca) molar ratios of 0 C.HA/COL + Zn Zn/(Zn + Ca) molar ratios of 0.05		µ-CT H&E	BV/TV A:22.87%±4.46 B:40.18%±4.41% C: 76%±4.46% NBF A: 13.16%±3.92% B: 32.55%±5.66% C: 42.27%±5.74%	(52)
Begam (2017)	Zn	HA + Zn OR HA/COL + Zn + BMP2	Wet chemical method	-	male & female white New Zealand rabbits	4x5 mm Femoral defect	12W	A. Zn + HA B. Zn + HA + BMP2 C. Zn + HA /COL + BMP2		H&E SEM IHC H&E of liver and kidney (toxicology)	NBF: B > C Kidney and liver normal	(53)
Chou (2013)		Zn-TCP	hydrothermal conversion	-	male wistar rat	2x3 mm tibial defect	8 W	A. Zn-TCP B. βTCP C. Empty		µ-CT H&E CT	faster rise in bone mineral density in A compared to B or C but BMD at 8^th^ week: A=B restoration of the trabecular bone in the A was more mature and dense compared with B both A & B were able to stimulate regeneration of new bone closing the defect, but were also regenerating cancellous bone	(65)
Kawamura (2003)		TCP+ Zn + HA	Sol-Gel method	-	New Zealand White rabbits	Transcortical Femoral defect	2-60 W	A. ZnTCP, TCP, and HAP powders B. TCP/HAP composite ceramic		IHC	The Change in cortical bone apposition rate (C-BAR) 24^th^ week A: 85.3 ± 6.0% B: 70.8 ± 6.9% C-BAR 60^th^ week A: 74.5 ± 17.5% B:52.6 ± 10.3% Change in intramedullary bone apposition rate (IM-BAR) 6^th^ week A: 61.4 ± 26.7% B: 42.6 ± 18.5% Medullary cavity area (MCA) A: 15.7 ± 3.9 B: 12.2 ± 3.7	(55)

**Table 2:** Strontium in bone tissue engineering

Author (year)	Scaffold	Fabrication method	Cell type	Animal model	Defect size & location	Implantation period	Groups	Parameters	Observed effect	Ref
Liu (2019)	PCL + HA + Sr	3D printing	-	SD rats	5 mm critical Size calvarial defect	12 W	A. PCL B. PCL + HA C. PCL + HA + Sr	µ-CT H&E MT	NBF: C>B>A	(36)
Prabha (2019)	PCL + SRA	Solution blending method	human telomerase immortalized bone marrow derived keletal stem cell line (hMSC-TERT)	NOD.B17-Prkdcscid/J mice	Subcutaneous implantation	8 W	A.PCL + cell B. PCL + SRA + cell C. PCL D.PCL + SRA	H&E HVM Sirius red	matrix was rich in Type I collagen areas of vascularized ectopic bone matrix formation was seen	(37)
Lino (2018)	PCL + PDIPF + 1% or 5% Sr	Solvent casting	-	WKAH/Hok Wistar rats	2 mm calvarial defect	4W	A.PCL/PDIPF B. PCL/PDIF + 1% Sr C. empty	H&E qRT-PCR	NBF: B= A + 52% Runx2: C>B>A	(38)
Han (2019)	PLLA + Sr	electrospinning	-	SD rat	5 mm critical size calvarial defect	8W	A. PLLA B. PLLLA C. PLLLA + 5 Sr D. PLLLA + 10 Sr E. PLLLA + 15 Sr	µ-CT H&E MT Gieson IHC	NBF: E>D>C>B>A CA A:0.297 ± 0.085% B:0.657 ± 0.17%) C: 1.26 ± 0.079% D:1.37 ± 0.085% E: 1.657 ± 0.13%, AR: A:0.397±0.148%, B:0.793±0.194%, C:1.133±0.1%, D:1.647±0.107% E:1.97±0.123% 0.05 TE: A:0.277±0.1% B:0.337±0.08% C:0.737±0.102% D:1.337±0.111% E:1.427±0.135%	(39)
Yan	HA + PBLG +Sr	coprecipitation + aminated surface modification	Rabbit ADSCs	New Zealand rabbit	Critical full-thickness segmental Femoral defect	12, 24 W	A. HA + PBLG +Sr B. HA + PBLG +Sr + ADSCs	µ-CT H&E MT	B>A	(40)
Ge (2018)	PLLA + HA + Sr	lyophilization	-	JW rabbits	3.5 mm femur defect	5W	A. PLLA B. PLLA + HA C. PLLA + HA + Sr	µ-CT H&E MT	NBF : C>B>A=0	(41)
Yu (2017)	APMs + COL + Sr	Microwave-Hydrothermal Method	-	SD rat	5mm critical size calvarial defects	8 W	A. Col B. APMs/col C.SrAPMs/col	µ-CT H&E IHC	BMD: A: 55.88 ± 14.89 B: 213.12 ± 52.05C: 334.78 ± 41.08 BV/TV: A: 3.36 ± 0.76% B: 20.64 ± 7.33% C: 48.30 ± 11.75% NBF: C>B>A OCN & OPN: C>B	(12)
Author (year)	Scaffold	Fabrication method	Cell type	Animal model	Defect size & location	Implantation period	Groups	Parameters	Observed effect	Ref
Gao (2017)	HA + PBLG + Sr	covalent surface functionalization	Mouse ADSCs	C57BL6/J mice	2mm critical size in middle femur	8W	A. HA + PBLG + Sr B. HA + PBLG + Sr + ADSCs	x-ray radiography µ-CT H&E	NBF: B>A BV/TV: B= normal bone > A	(42)
Wang (2016)	SCPP	gravity sintering	-	New Zealand white rabbits	10mm critical size calvarial defects	4,8,12 W	A. Sr.SCPP B. DOPA/Sr.SCPP C. DOPA/Sr.SCPP/Silk fibroin	H&E IHC	NBF Max in C VEGE and bFGF Max in C	(43)
Xie (2013)	SCPP	Gravity sintering	-	New Zealand white rabbits	15 x 5 mm defect above thighbone	12 W	A. HA B. CPP C. SCPP	H&E IHC x-ray microradiography	NBF: C>B>A VEGF: C>B>A	(44)
Gu (2013)	SCPP		Rabbit BMSCs	New Zealand white rabbits	15 mm segmental radius defect	4,8,16 W	A. HA B. CPP C. SCPP D.HA + MSCs E.CPP + MSCs F.SCPP + MSCs	H&E MT Toluidine blue x-ray microradiography	NBF 4^th^ week D<E or F C,D,E,F significantly higher than A NBF 16^th^ week F>E>C>B>D>A NBF in x-ray Max in F	(45)
Yang (2011)	HA + 10% Sr	Hydrothermal method	-	Female SD rat	5mm critical size calvarial defects	4, 12 W	A. COLB. COL + HA C. COL + HA + Sr	H&E MT CT	HU 1^st^ month: A: 15.7 ± 3.3 B: 121 ± 14.2 C: 219.4 ± 37.1 HU 3^rd^ month: A: 59.4 ± 8.5 B: 249.2 ± 20.5 C: 381.7 ± 25.3 NBF: B: 0.93 ± 0.07 C: 1.48 ± 0.13 Mature bone area (fold increase compared to A): B: 11.6 ± 0.6 C: 3.4 ± 0.7 β-catenin, OPN, COL1 C>B	(46)
Li (2010)	HA + Col + Sr	Sol-Gel	-	SD ovareictomized rat	1 mm intercondylar channel into medullary canal in tibiae	12 W	A.HA B.HA + Sr	µ-CT Toluidine blue	Bone area ratio: B= A + 70.9% Bone to implant contact: B=A + 49.9% BV/TV: A:24.7 ± 4.9 B: 42.9 ± 6.7 B=A + 73.7%	(47)

APMs: amorphous calcium phosphate porous microspheres; HVM: human Vimentin antibody; PBLG: poly(γ-benzyl-L-glutamate); PDIF: polydiisopropyl fumarate; rBMSC: rat bone marrow derived stem cells; SCPP: strontium doped calcium polyphosphate; Sr: Strontium; SRA: laponite-strontium ranelate.

## Discussion

Biomimetic bone scaffolds incorporating additional therapeutic agents like MITAs are a main focus of BTE. The specific biological advantage that these ions bring to scaffolds as well as other potential mechanical, and antimicrobial enhancements may vary depending on the ion entity, fabrication method, and biomaterials used. Herein, we categorized in-vivo studies on MITAs in bone substitutes with the aim of clarifying their efficacy and identifying the affecting parameters. 

The most frequently used ion, strontium (Sr), is a naturally occurring ion with 98% of it localized in the skeleton, exchanged with Ca^2+^ in the HA crystal lattice (3). Sr^2+^, a structurally similar ion to calcium, helps promote osteogenic differentiation of mesenchymal stem cells (MSCs) via wnt/βcatenin and Ras/MAPK signaling pathways (46) and inhibits osteoclastic activity. Strontium has thus been widely investigated in both *In-vitro* and *In-vivo* studies and has been shown to enhance NBF, remodeling and ossseointegration when added alone to scaffolds or combined with other ions (41, 66-68). However, high doses of Sr have been shown to have adverse effect on calcium absorption and bone mineralization, therefore, engineering a controlled release scaffold is of great importance (69). In our review, Sr enhanced NBF in nine studies while two studies had no control scaffolds without Sr to make the comparison possible. One study failed to report any significant difference between laponite-strontium ranelate containing PCL and PCL alone with or without cells (37) which could be due to ectopic implantation. Gao et al., reported bone volume/total volume (BV/TV) resembling that of natural bone using Sr-HA-graft-poly(benzyl-L-glutamate) nanocomposite microcarriers loaded with ADSCs in a mice 2mm critical-sized femoral defect model (42). 

Magnesium was also extensively studied in combination with PLGA and PCL polymers and HA. Mg^2+ ^is another element found in human body, half of which is deposited in bone tissue (70). Studies have shown a correlation between magnesium deficiency and osteoporosis, attributable to changes in parathyroid hormone (PTH), Vitamin D levels and increased pro-inflammatory cytokine secretion such as substance P, TNF-α, IL-1β, and RANKL (71). Similar to strontium, magnesium works by stimulating MSCs proliferation, differentiation while suppressing osteoclast activity. Moreover, it has been demonstrated that magnesium increases osteogenic gene expression and protein expression of collagen type X and VEGF (72). Additionally, magnesium is reported to have antibacterial properties, beneficial in reducing infection risk in bone grafting procedures (73). Almost all studies reviewed in this article, proved magnesium efficient in promoting NBF with the exception of a study by Suryavanshi et al. that reported no NBF using PCL and MgO in subcutaneous implantation. The scaffold, however, was proved to be biocompatible (48). In another study by Deng et al. in a goat bi-parietal 15 mm calvarial defect model, the synergistic effect of magnesium and human recombinant bone morphogenetic protein 2 (rh-BMP2) was also demonstrated (49).

Another frequently studied ion is zinc, a trace element essential for neural growth, immunological functions and many other cellular processes (74). Zinc is well recognized as a critical mineral for bone health and development, as its deficiency is associated with bone growth lag and mal-development as well as osteoporosis (75). Zn^2+^ affects MSCs through ERK1/2 signaling and hinders osteoclasts by antagonizing NF-κB pathway (76). Zinc also processes antibacterial effects owing to production of reactive oxygen species (ROS) and aids in wound healing (77). Of the five *In-vivo* studies reported in this study, one study provided no control for comparison (53), while another study showed that both βTCP and Zn-TCP were able to stimulate regeneration of new bone closing the defect but Zn-TCP showed a faster rise in bone mineral density and resulted in a more mature and denser trabecular bone (54). A long-term (60 weeks) study on bone formation in white New Zealand rabbit transcortical femoral defect model also presented that Zn-HA-TCP was able to promote NBF compared to HA-TCP (55). Similar results were obtained in an 8-week study on Zn-HA-Collagen in SD rat model (52). Samanta et al. conducted a study in a rabbit femoral defect model where TCP-Zn was compared to TCP-Ti and TCP-Mg. NBF was increased to 65.06 ± 3.0 in TCP-Ti, compared to 50.55 ± 2.0 in TCP-Mg, 48.40 ± 2.0 in TCP-Zn, and 34.24 ± 2.0 in TCP alone (22). More similar studies are recommended to make the comparison between various ions possible.

Data on other less commonly applied metallic ions can be found in Table 1. The readers are referred to two published narrative reviews on metallic ions for further explanation of the functions of these ions (3, 72). Undoubtedly, the most frequently used ion in bone structure and bone substitutes is calcium. However, as their obvious role in bone tissue regeneration has been well documented, the authors agreed to limit this systematic review on ions, whose potential role and efficacy are yet to be determined. The authors also excluded studies on mixtures of ions and alloys as such studies would not help in drawing conclusions regarding the efficacy of a specific ion. There is a body of *In-vivo* literature on magnetic scaffolds, of which only the ones that housed animals in normal cages are included. Studies stimulating bone formation by creating a magnetic field around the animal during implantation period were excluded because it was agreed that the underlying mechanism is rather different than that of MITAs. The readers are kindly encouraged to read a review by Xu et al. on magnetic responsive scaffolds in BTE (78).

As presented in table 1, various polymers, ceramics and composites are functionalized using MITAs. Among the polymers, PCL seems to be more frequently used which could be explained by its relative simple application in the fabrication of scaffolds. PCL is a non-immunogenic synthetic polymer which can be dissolved in most of organic solvents, there are many methods to its fabrication and it can be blended with other polymers or ceramics to hand in composite scaffolds with enhanced mechanical properties especially in load bearing areas. The slow degradation rate and unfavorable water contact angle are among its drawbacks (79). Hydroxyapatite is the most applied ceramic doped with MITAs in our review. HA is a calcium phosphate similar to that of human hard tissues in morphology and composition with an identical Ca/P ratio to bone apatite. HA has been extensively used in BTE due to its stability in physiological conditions, biocompatibility, osteoinductivity and non-toxicity and non-inflammatory nature (80). In our review, HA was introduced into animal defects alone or with natural or synthetic polymers such as collagen, PLLA, PLGA and chitosan. Such composite scaffolds have the advantage of making the scaffold osteoconductive and reinforcing the mechanical characteristics of scaffold further mimicking natural bone architecture (81). The efficacy of MITAs in improving bone tissue regeneration was evaluated in mesoporous bioactive glasses (MBG) by some researchers. We excluded all these studies because as explained before, the specific influence of an ion could be obscured by the synergistic effect they may have with other ions in MBG. There is a valuable review on MBG incorporating MITAs for bone tissue engineering which can be considered in addition to the present study (82).

A meta-analysis by shanbhag et al. indicated statistically signiﬁcant beneﬁts in loading scaffolds with cells with a weighted mean difference of NBF of 15.59–49.15% and 8.60–13.85% NBF in large- and small-animal models, respectively (83). In our review, seven studies seeded the scaffolds with various types of cells. In one study, hypoxia preconditioned BMSCs enhanced NBF more than normal BMSCs and they both scored higher NBF compared to unloaded HA + Li scaffold (8). Wang et al. and Gu et al. loaded all scaffolds with cells and prabha et al. reported no enhancement of NBF when loading scaffolds with hBMSCs (10, 37, 84). Yan et al. and Gao et al. also showed improved NBF using rabbit and mouse ADSCs (40, 42). 

Animal models play a crucial role in testing bone scaffolds for understanding their osteoconductivity, biocompatibility, mechanical properties, degradation, and interaction with host tissues (85). Small animal models used in BTE research are primarily rodents (rats and mice) and rabbits (83). In our review, almost all studies used small animals because of significantly lower costs, and easier housing and handling (86). Rodents also have a less varied genetic background in terms of biological response which makes the statistical analysis credible (87). However, before generalizing results from these studies to humans, the differences in the structure and composition of these animals’ bones as well as faster skeletal change and bone turnover in these animals should be considered (88). Another limitation is the inability to create multiple defects to study different materials simultaneously (89). In two studies, larger animals, namely beagle dogs and goats were used. Dogs are widely used in musculoskeletal research, given the similarities in structure and physiology of canine and human bone. However, limitations of ethical issues, high costs, handling difficulties exist (89). One study used normal human osteoblast cell line, claiming that it mimics the cellular event of the *in-vivo* intramembranous bone formation process and reported the study as an *In-vivo* model which was excluded by the agreement of authors (90).

Critical-sized defects were created in these animal models in order to observe osteoinductive capacity of scaffolds incorporating MITAs. A critical-sized defect is defined as “the smallest osseous wound that does not heal spontaneously over a long period of time or more clinically relevant, that which has no mineralized area ˃ 30% after 52 weeks” (85). For the rat calvarial defect, 8 mm is generally reported to be the critical size; however, smaller defects have been investigated in models with bi-parietal defects, resulting in fewer sacrificed animals (91). In our review, nine articles regarded 5 mm defects as critical which is suggested to be replaced with 8 mmm defects in future studies. Three studies evaluated the newly designed scaffolds in subcutaneous implantation, two of which could not report NBF (10, 37, 48). Therefore, it is suggested that more accurate defect models be used in BTE researches. 

The most frequently conducted test to evaluate I*n-vivo *bone formation were µ-CT, and H&E staining. The biological performances of a scaffold regarding cell adhesion, proliferation and mineral deposition as well as formation of mature bone with vasculature are factors to be evaluated during *In-vivo* implantation testing (92). µ-CT is a non-destructive computational technique capable of providing 3D images of engineered constructs as well as quantitative data based on the fact that new bone, fibrous tissue and scaffolds have different coefficients of absorption (93). Studies have also focused on other aspects of bone substitutes containing MITAs such as in tumor suppression capability, angiogenetic ability, immune response induction, etc. (94-96). However, all the articles not evaluating the formation of new bone were excluded in our study because they were not consistent with the aims of this review.

*Limitations*

Few studies did not have the control group, and several variations including cell sources, scaffold types, fabrication methods, and measured parameters between included studies do not permit general conclusions to be drawn. 

## Conclusion

A systematic review on *In-vivo *studies on MITAs used in Bone tissue engineering showed several important findings: 1) various materials can be successfully used to incorporate MITAs and one must opt for the composition that renders the best biological response as well as physicochemical characteristics. 2) Of the various fabrication methods applied in BTE for integrating MITAs into scaffolds, it is important to consider their effect on controlled release of ion besides the ease of method as many ions can have deleterious effects if the therapeutic doses are surpassed. 3) A tendency to enhance new bone formation with the use of MITAs can be observed in the studies. However, this needs to be validated with further studies comparing various ions with each other and the different concentration in the same animal model using critical-sized defects. 

report.
